# PROS1 shapes the immune-suppressive tumor microenvironment and predicts poor prognosis in glioma

**DOI:** 10.3389/fimmu.2022.1052692

**Published:** 2023-01-04

**Authors:** Jinxiang Wang, Nisha Wu, Xiaowei Feng, Yanling Liang, Meijin Huang, Wenle Li, Lingmi Hou, Chengliang Yin

**Affiliations:** ^1^ Academician (expert) workstation, Sichuan Key Laboratory of Medical Imaging, Breast Cancer Biotargeting Laboratory, Affiliated Hospital of North Sichuan Medical College, Nanchong, Sichuan, China; ^2^ Department of Cell Biology, School of Basic Medical Sciences, Southern Medical University, Guangzhou, China; ^3^ Department of Clinical Laboratory, The Fifth Affiliated Hospital, Southern Medical University, Guangzhou, China; ^4^ Department of NeuroRehabilitation, Shaanxi Provincial Rehabilitation Hospital, Xi’an, China; ^5^ Department of Clinical Laboratory, Affiliated Foshan Maternity and Child Healthcare Hospital, Southern Medical University, Foshan, China; ^6^ Department of Oncology, 920th Hospital of People’s Liberation Army (PLA) Joint Logistics Support, Kun ming, Yun nan, China; ^7^ State Key Laboratory of Molecular Vaccinology and Molecular Diagnostics and Center for Molecular Imaging and Translational Medicine, School of Public Health, Xiamen University, Xiamen, China; ^8^ Faculty of Medicine, Macau University of Science and Technology, Macau, Macau SAR, China

**Keywords:** PROS1, exhausted T cell, biomarker, glioma immunosuppressive microenvironment, prognosis

## Abstract

**Background:**

Glioma is the most malignant cancer in the brain. As a major vitamin-K-dependent protein in the central nervous system, PROS1 not only plays a vital role in blood coagulation, and some studies have found that it was associated with tumor immune infiltration. However, the prognostic significance of PROS1 in glioma and the underlying mechanism of PROS1 in shaping the tumor immune microenvironment (TIME) remains unclear.

**Methods:**

The raw data (including RNA-seq, sgRNA-seq, clinicopathological variables and prognosis, and survival data) were acquired from public databases, including TCGA, GEPIA, CGGA, TIMER, GEO, UALCAN, and CancerSEA. GO enrichment and KEGG pathway analyses were performed using “cluster profiler” package and visualized by the “ggplot2” package. GSEA was conducted using R package “cluster profiler”. Tumor immune estimation resource (TIMER) and spearman correlation analysis were applied to evaluate the associations between infiltration levels of immune cells and the expression of PROS1. qRT-PCR and WB were used to assay the expression of PROS1. Wound-healing assay, transwell chambers assays, and CCK-8 assays, were performed to assess migration and proliferation. ROC and KM curves were constructed to determine prognostic significance of PROS1 in glioma.

**Results:**

The level of PROS1 expression was significantly increased in glioma in comparison to normal tissue, which was further certificated by qRT-PCR and WB in LN-229 and U-87MG glioma cells. High expression of PROS1 positively correlated with inflammation, EMT, and invasion identified by CancerSEA, which was also proved by downregulation of PROS1 could suppress cells migration, and proliferation in LN-229 and U-87MG glioma cells. GO and KEGG analysis suggested that PROS1 was involved in disease of immune system and T cell antigen receptor pathway. Immune cell infiltration analysis showed that expression of PROS1 was negatively associated with pDC and NK CD56 bright cells while positively correlated with Macrophages, Neutrophils in glioma. Immune and stromal scores analysis indicated that PROS1 was positively associated with immune score. The high level of PROS1 resulted in an immune suppressive TIME *via* the recruitment of immunosuppressive molecules. In addition, Increased expression of PROS1 was correlated with T-cell exhaustion, M2 polarization, poor Overall-Survival (OS) in glioma. And it was significantly related to tumor histological level, age, primary therapy outcome. The results of our experiment and various bioinformatics approaches validated that PROS1 was a valuable poor prognostic marker.

**Conclusion:**

Increased expression of PROS1 was correlated with malignant phenotype and associated with poor prognosis in glioma. Besides, PROS1 could be a possible biomarker and potential immunotherapeutic target through promoting the glioma immunosuppressive microenvironment and inducing tumor-associated macrophages M2 polarization.

## Introduction

Glioma is one of the most common primary tumor affecting central nervous system, counting for 81% of all malignant brain tumors (1). The National Comprehensive Cancer Network (NCCN) Guidelines discovered glioma were heterogeneous groups of neoplasms, which range from surgically curable pilocytic astrocytomas of low-grade glioma (LGG) to highly invasive and virtually incurable glioblastoma multiforme (GBM) (2). Malignant glioma is among the most lethal adult cancers with five-year survival rate less than 3% (3). Although standard treatments such as surgical operation, radiation, or chemotherapy have increased recently, the prognosis of glioma remains very dismal. At present, immunotherapy has been a significantly promising treatment in malignant tumors, mainly including immune checkpoint inhibitors, peptide vaccines, and CAR-T cells (4). However, high-grade glioma, called largely immunologically “cold” tumor, was characterized by an immunosuppressive tumor microenvironment with few tumor-infiltrating T cells and resistance to checkpoint inhibitors, further leading to poor prognosis (5, 6). Notably, the Blood-brain barrier (BBB), a special physiological structure of the brain, reduces the efficiency of tumor immunotherapy (7). Nevertheless, the known molecular biomarkers are inadequate to reflect individual heterogeneity and reveal the possible glioma risks. Thus, it is of great significance to find a new prognosis and immunotherapy molecular marker in glioma.

Protein S (PROS1) is a recognized ligand of the Tyro3, AXL, and Mer (TAM) family of tyrosine kinases receptors, it was secreted by activated T cells (8) or tumor-associated macrophages (9). PROS1 is exposed on the surface of apoptotic cells to induce an inflammatory response by activating TYRO3 and MERTK (8). Meanwhile, PROS1 is vitamin K–dependent plasma protein playing a vital role in physiologic anticoagulation (10), glomerular injury, and periodontitis (11, 12). In addition, PROS1 plays two roles as both an anticoagulant and a proto-oncogene. Ichiro Nakano et al. reported that the PROS1 promotes tumor development through the AXL pathway in aggressive GBM tumors (13). Moreover, H Shelton Earp et al. found PROS1 could effectively inhibit expression of macrophage M1-associated genes *via* Mer and Tyro3 (14). In recent years, PROS1 has been proven to be a potential target in several cancers, such as breast cancer (15), bladder cancer (16), oral squamous cell carcinoma (17), and aggressive prostate cancer (18). Yet, the comprehensive understanding of PROS1 on its expression level, clinical prognostic value, and its underlying mechanisms in gliomas is still unclear. It is crucial for us to explore the fundamental roles and mechanisms of PROS1 in tumor progression and the correlation between PROS1 expression and immune microenvironment in glioma.

In our study, through online database analysis, it was found that PROS1 expression level was significantly increased in glioma, and its high-expressions in LN-229 and U-87MG glioma cells were identified through the qRT-PCR and WB assay. *In vitro* experiments, knockdown of PROS1 could inhibit cells migration and proliferation of LN-229 and U-87MG glioma cells. Furthermore, we used single -cell analysis, GSEA, Go test and KEGG pathway analyses to investigate the roles of PROS1 in glioma, found that PROS1 was involved in the disease of the immune system and T cell antigen receptor pathway. Then, the relationship between PROS1 expression and immune micro-environment, immune examination point inhibitors, immune cell markers and treatment results were exhaustively analyzed. The results showed that PROS1 promoted the malignant progress of glioma by shaping the micro-environment of immunosuppressive tumors, such as T cell failure, immunosuppressive cell infiltration, and the polarization of M1-type macrophages to M2 -type macrophages. This work emphasizes that PROS1 may be a promising target for immunotherapy.

## Materials and methods

### Data download and collation

The glioma RNA sequencing (RNA-seq) data (a total of 696 cases, including GBM and LGG) and clinical information were obtained from the TCGA website (http://www.tcga.org/). We performed the HPA database (https://www.proteinatlas.org/) to demonstrate PROS1 protein expression among normal and cancer tissues. We acquired the immunohistochemistry results of PROS1 proteins in normal tissue and glioma patients. In addition, 5 tumors and 17 normal, 40 tumor and 6 normal samples were downloaded from two GEO microarray datasets (GSE42656 and GSE22866).

### Protein level analysis

The Human Protein Atlas (HPA) (https://www.proteinatlas.org/) database was employed to investigate the protein level of PROS1 in human tumor and normal tissues.

### CGGA mRNA matrix and clinical information

Total 1018 glioma samples were obtained from the Chinese Glioma Genome Atlas (CGGA) (http://www.cgga.org.cn), the largest glioma genome database in China (19). All the glioma samples and clinical information were acquired with informed consents. The survival rates in the PROS1 expression of this data were analyzed. What’s more, the mRNA-seq_325 dataset was downloaded as well for further study. We listed the survival and gene expression of PROS1 using R software.

### UALCAN analysis

UALCAN (http://ualcan.path.uab.edu/analysis-prot.html) (20) is an online tool that provides comprehensive analyses of transcriptome data from The Cancer Genome Atlas (TCGA). We conducted a CPTAC dataset to assess protein levels of PROS1 between normal tissues and primary tumor tissues.

### Analysis of DEGs between PROS1-high and -low expression glioma groups

Unpaired Student’s t-test was used to identify DEGs between PROS1-high and PROS1-low groups from TCGA datasets within the DESeq2 (3.8) package (21). Genes with the adjusted *P*-value <0.05 and absolute FC > 2 were considered as statistically significant. All DEGs were represented in heat map.

### Cell culture

Human Glioma cancer cell lines LN-229 and U-87MG were purchased from ATCC. The rest cells were maintained in DMEM medium supplemented with 10% FBS (Excell, FSP500), 100U/mL streptomycin, and 100U/mL penicillin. Cells were cultured in incubator containing 5% CO2 at 37°C.

### *In vitro* siRNA treatment

Glioma cancer cells were transfected with non-silencing scrambled control siRNA (NC) and human small interfering RNA (siRNA). The siRNAs were designed and synthesized by SyngenTech Company (Beijing, China). Transfection was conducted with Lipofectamine 3000 transfection reagent (Invitrogen, 2319757, USA) following the manufacturer’s instructions. 48h after transfection, cells were collected and subjected to other experiments. The two small interfering RNA (siRNA) sequences targeting PROS1 (Gene cluster ID:5627) used were as follows: NC (Sense:5′-UUCUCCGAACGUGUCACGUTT-3′; Anti-sense:5′-ACGUGACACGUUCGGAGAATT-3′); si-1(Sense:5′-GCGUGAUACUGUACGCAGATT-3′; Anti-sense:5′-UCUGCGUACAGUAUCACGCTT-3′); si-2(Sense:5′-GAGUUGUCGACACCACUUATT-3′; Anti-sense:5′-UAAGUGGUGUCGACAACUCTT-3′).

### Western blot

Total protein was extracted in RIPA lysis and extraction buffer (Aidlab Biotechnologies Co, Ltd, USA) and separated by 10% SDS-PAGE. The separated proteins were transferred to a PVDF membrane (IPVH00010). The results were visualized by Goat anti-Mouse IgG(H+L)-HRP (Beijing Ray Antibody Biotech, RM3001) and Goat anti-Rabbit IgG(H+L)-HRP (Beijing Ray Antibody Biotech, RM3002, China). The antibodies were as follows: GAPDH Mouse mAb (1:1000, Abclonal, AC002, China) and PROS1 Rabbit pAb (1:1000, Abclonal, A1595, China).

### Quantitative real-time polymerase chain reaction (qRT-PCR)

Total RNA was extracted by Trizol reagent (Invitrogen) and the cDNA was reversely transcribed from total RNA by RT SuperMix (Vazyme, R222-01. According to manufacturer’s instructions, Quantitative real-time PCR was performed by ChamQ/SYBR qPCR Master Mix (Vazyme, Q311-03) and the 7500 Fluorescent Quantitative PCR System (Applied Biosystems Life Technologies, USA), using the thermal cycling profile as: 95°C, 3 minutes; 40 cycles — denaturation at 95°C, 10seconds, annealing at 60°C, 30 seconds, extension at 72°C, 35seconds. The ΔΔCT method was conducted to analyze results and GAPDH was employed as internal control. The detailed primer sequences are as follows: GAPDH forward:

5′- GTCTCCTCTGACTTCAACAGCG′ and Reverse: 5′-ACCACCCTGTTGCTGTAGCCAA-3′;

PROS1 Forward: 5′- GGCTCCTACTATCCTGGTTCTG′ and Reverse:

5′- CAAGGCAAGCATAACACCAGTGC-3′

### Wound healing assay

Migration ability of cancer cells was evaluated with wound healing assay. When the cells took up 95% area of the 6-well plate bottom, the monolayer was scraped with a 200 μL pipette tip. Then, glioma cells were rinsed with phosphate buffer and starved to migrate for the indicated time (0, 12, and 24 h). At last, cell migration was observed under optical microscope, and distance of migration was calculated by ImageJ soft The scale bar in the representative scratch images was 0.5 mm.

### Transwell migration analysis

Cell migration were further analyzed with transwell chambers (8 μm pore size, Corning, NY, United States). Preparation of cell suspension for transwell migration experiment: glioma cells were cultured for 12 h in serum-free DMEM medium to remove serum influence. Then, the density was adjusted to 5× 10^4^ cells/mL in the upper chamber with serum-free medium, and DMEM medium with 10% FBS was added into the lower chamber. Cotton swabs were used to wipe off the cells on the top side of the upper chamber after 48 hours of culture. The cells that migrated to the bottom surface were fixed with 4% paraformaldehyde and stained with 1% crystal violet.

### Cell counting kit‐8 (CCK‐8) assay

Cells proliferation was evaluated with CCK 8 assay. A density of 2000 cells/well was seeded in a 96-well plate and three parallel wells were set for each group. After 1, 2, 3, and 4 days of culture, 10 µL CCK-8 reagent (Dojindo, CK04, Japan) and 100 µL DMEM medium were added into glioma cancer cell lines. Then, the cells were incubated for 1 h at 37°C and absorbance were measured at 450 nm.

### Functional enrichment and analysis of immune cell infiltration

Differential analysis was performed using the limma package (22). DEGs between PROS1-high and PROS1-low groups from TCGA datasets were identified by the unpaired Student’s t-test, within the DESeq2 (3.8) package. Gene ontology (GO) enrichment and KEGG pathway analyses of co-expression genes were demonstrated by the “ClusterProfiler” package (23) and visualized by the “ggplot2” package. The relative tumor infiltration levels of 24 immune cell types were quantified by ssGSEA to interrogate expression levels of genes in published signature gene lists (24). The relationship between PROS1 expression and infiltration levels of immune cells was analyzed with Wilcoxon rank-sum test and Spearman correlation.

### TIMER database analysis

TIMER is an online tool (https://cistrome.shinyapps.io/timer/) (25). It was employed to investigate the correlation of PROS1 expression with immune cell infiltration level in glioma. *P*-value <0.05 was considered as statistically significant.

### Drug sensitivity analysis

The drug sensitivity analysis was evaluated using the GSCA (26), GDSC, and CTRP databases.

### Statistical analysis

Most statistical analyses were conducted using R language, version 3.6.16, and the others were with GraphPad Prism 8.0 and SPSS 22 software. The association between PROS1 expression levels and clinicopathological features was analyzed with Chi-square test. The Cox proportional hazards regression model was conducted for multivariate and univariate analyses to explore the independence of PROS1 in predicting clinical prognosis. Kaplan-Meier analysis was used to find out the survival rate with cox regression test. Spearman’s correlation coefficients were applied to evaluate the correlation between PROS1 expression and marker genes of immune infiltrating cells. All statistical analyses were two-sided, and *P* < 0.05 was considered to be statistically significant.

## Results

### High expression of PROS1 in glioma

**Figure 1:**
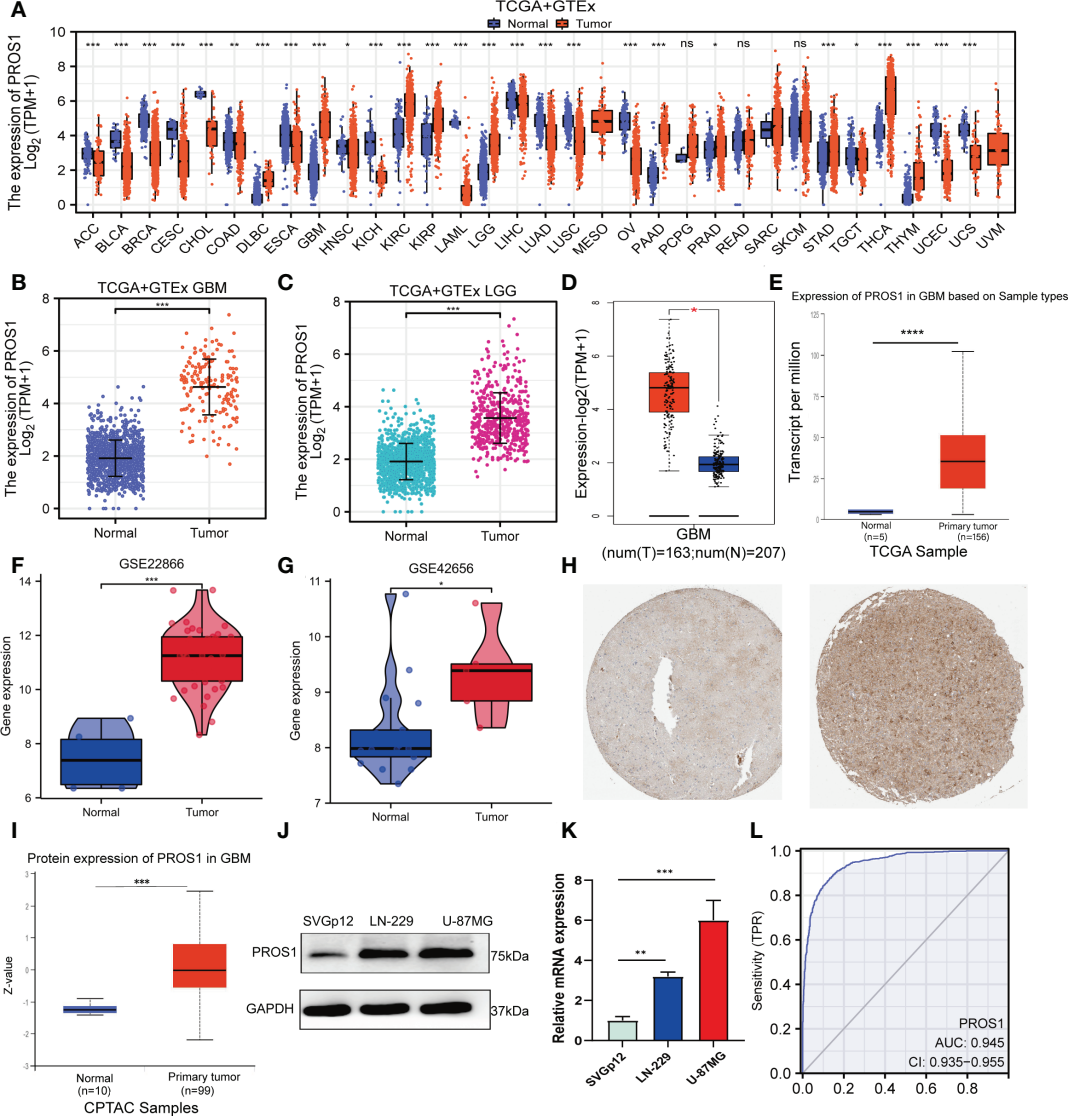
PROS1 expression levels between human cancer and normal tissues. **(A)** Pan-cancer analysis of PROS1 expression across all tumor samples and normal tissues from TCGA and GTEx database. **(B, C)** The PROS1 expression levels between tumor and normal tissues in GBM and LGG from TCGA and GTEx database. **(D, E)** The PROS1 expression levels between tumor and normal tissues in GBM from GEPIA and UALCAN. **(F, G)** The PROS1 expression levels between glioma and non-cancer tissues form GSE22866 and GSE42656. **(H)** Immunohistochemistry of PROS1 expression in the glioma tissue microarray cohort in the HPA database. **(I)** The protein expression of PROS1 between normal tissues and primary tumor tissues in the CPTAC dataset. **(J, K)** Western blotting and qRT-PCR assays the protein and mRNA expression of PROS1 in two tumor cell lines and one normal cell line (LN-229, U-87MG, and SVGp12). **(L)** ROC analysis of PROS1 expression shows promising discrimination power between glioma and non-tumor tissues. (ns, nonsignificant; *P* > 0.05; *, *P* < 0.05; **, *P* < 0.01; ***, *P* < 0.001; ****, *P* < 0.0001).

First, the pan-cancer analyses results showed that the expression of PROS1 was increased in different kinds of tumors, including GBM and LGG (*P <*0.001) (**Figure 1A**). Besides, we further found that PROS1 expression in 166 tumor tissues was higher than in 1157 normal samples in GBM (*P* < 0.001), and expression of PROS1 in 523 tumor tissues were higher than that in 1152 normal samples in LGG (*P <*0.001) (**Figures 1B, C**). Consistently, we also found that PROS1 expression was highly elevated in GBM tissues than in normal tissues in the GEPIA and UALCAN databases (**Figures 1D, E**). Besides, from two glioma studies of the GEO database (GSE22866, GSE42656), it’s found that PROS1 expression was significantly increased in glioma tissues than in normal tissues (**Figures 1F, G**). Also, the protein level of PROS1 in glioma samples was higher compared to that in normal samples in the HPA and CPTAC database (**Figures 1H, I**), and our western blotting and qRT-PCR assays results further demonstrated that PROS1 protein and mRNA expression were increased in U-87MG and LN-229 glioma cell lines than in SVGp12 normal cell line (**Figures 1J, K**). These results confirmed that PROS1 expressed higher in glioma.

The area under the curve (AUC) of PROS1is 0.945 (95% CI: 0.935–0.955) (**Figure 1L**) and the best cut-off value of PROS1was 2.654, suggesting PROS1 may be a potentially moderate identification molecule for glioma patients.

### Knockdown of PROS1 inhibits cell proliferation and migration of glioma cell line *in vitro*

**Figure 2:**
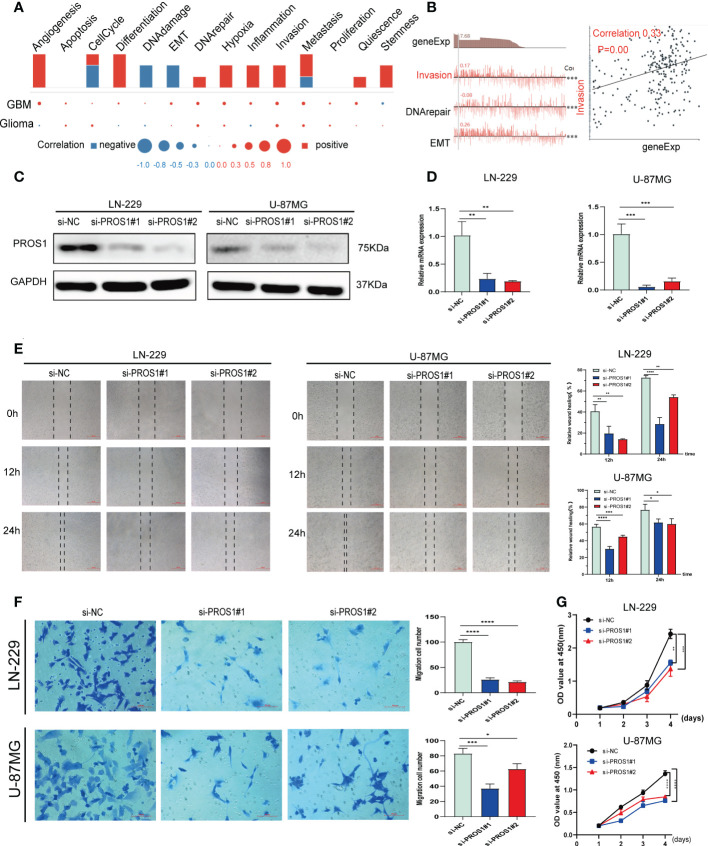
PROS1 increased proliferation, migration, and invasion of Glioma cells *in vitro*. **(A)** Single-cell analysis showed the functional state of PROS1 in Glioma. **(B)** CancerSEA analysis demonstrated that PROS1 positively correlated with invasion. **(C, D)** The western blotting and qRT-PCR were applied to analyze the protein and mRNA expression level of PROS1 after transfection by siPROS1 or PROS1 knockdown control for 24 h in LN-229 or U-87MG cells. Wound healing assays (scale bar: 500 μm) **(E)** and Transwell assays (scale bar: 100 μm) **(F)** were performed in transfected LN-229 or U-87MG cells to evaluate cell migration ability. **(G)** Cell proliferation of LN-229 or U-87MG cells after knocking down was determined by CCK8 assays. (*, *P* < 0.05; **, *P* < 0.01; ***, *P* < 0.001; ****, *P* < 0.0001).

To further explore the implications of PROS1 in glioma, a single-cell analysis conducted by using CancerSEA database. The CancerSEA analysis demonstrated that PROS1 positively correlated with inflammation, invasion, metastasis, and proliferation in glioma (**Figures 2A, B**). We further investigated whether PROS1 regulated the malignant phenotype of glioma. Therefore, we downregulated PROS1 expression using small interfering RNA in the two glioma cell lines (LN-229, U-87MG). the protein and mRNA expression levels of PROS1 significant decreased (**Figures 2C, D**). The wound-healing assays and transwell assays showed that PROS1 knockdown could suppress the migration ability of glioma cells (**Figures 2E, F**). The proliferative curves of CCK8 assays indicated a significant drop (*P*< 0.05) in the number of cells during PROS1 knockdown, indicated that cell proliferation was positively related to the expression of PROS1 in LN-229 and U-87MG cell lines (**Figure 2G**). These results suggested that PROS1 might participate in regulating the malignant progress of glioma.

### Identification of DEGs in glioma

**Figure 3:**
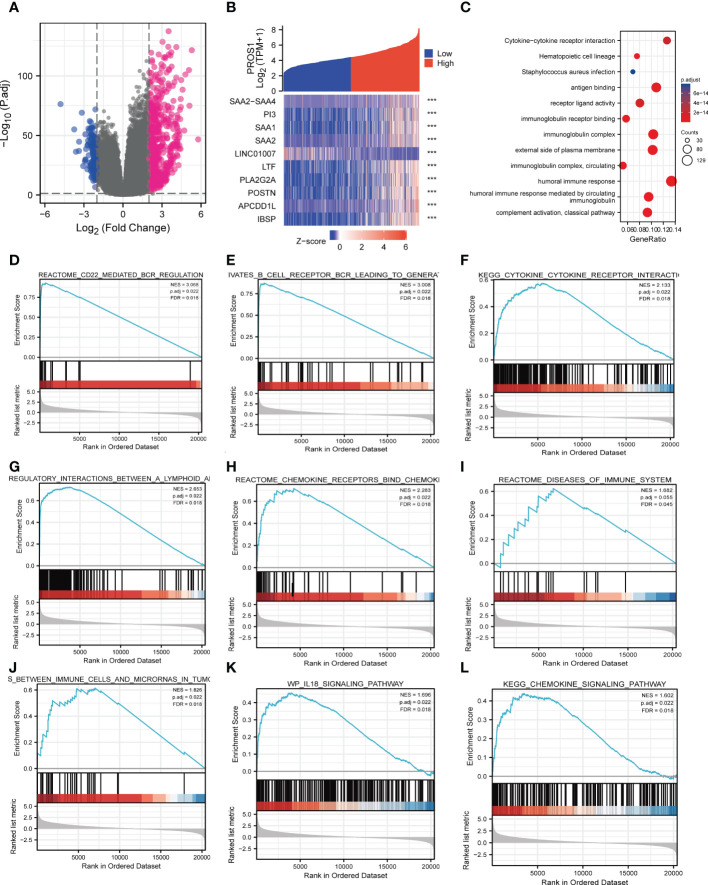
PROS1-related differentially expressed genes (DEGs) and functional enrichment analysis of high PROS1 expression glioma. **(A)** DEGs between glioma patients with high- and low- PROS1 expression. **(B)** Heat map of the 10 differentially expressed genes in the high- and low-PROS1 expression groups (***, *P* < 0.001). **(C)** GO and KEGG analysis of DEGs in glioma. Only pathways with a *P*-value <0.05 are presented. Enrichment plots from gene set enrichment analysis (GSEA). Several pathways and biological processes were enriched, including the **(D)** CD22 mediated regulation, **(E)** B cell receptor BCR, **(F)** cytokine receptor interaction, **(G)** regulatory interaction between A lymphoid, **(H)** chemokine receptors bind, **(I)** diseases of immune system, **(J)** immune cells and microRNAs in tumor, **(K)** IL18 signaling pathway, **(L)** chemokine signaling pathway. High and low expressions were defined as the PROS1 transcript level is more or less than the median level of all samples.

The HTSeq-Counts data from TCGA were analyzed applying the R package DESeq2 (adjusted *P* < 0.05, |log2 FC| > 2). As shown in the volcano plot, there were total of 763 DEGs, among them, 621 genes were positively correlated with PROS1 while 142 genes were negatively correlated (**Figure 3A**). 10 genes with differential expressions in the high- and low- PROS1 expression groups were illustrated in the heat map. (**Figure 3B**).

### PROS1 associated signaling pathways identified by GSEA

GO and KEGG pathway analyses were used to further analyze the biological enrichment process of 763 DEGs identified between the low- and high-PROS1expression groups, the results demonstrated that PROS1-related genes most significantly enriched in in many immune response-related processes, such as antigen-binding (*P* = 1.20e-82), immunoglobulin receptor binding (*P* = 8.08e-47), receptor-ligand activity (*P* = 1.25e-14), cytokine activity (*P* = 8.52e-13), immunoglobulin complex (*P* = 1.79e-93), humoral immune response (*P* = 7.46e-72), B cell-mediated immunity (*P* = 1.25e-68) (**Figure 3C**). In addition, the top 5 KEGG pathways for PROS1 and its correlated genes are shown in **Figure 3D**. Among these pathways, several immune-related pathways were highly associated with PROS1, such as cytokine-cytokine receptor interaction, Viral protein interaction with cytokine and cytokine receptors.

Significant differences (FDR < 0.25, adjusted *P-value* < 0.05) in the enrichment of several pathways in the MSigDB Collection (c2.cp.v7.2. symbols) were revealed. Among these pathways, many immune-related pathways were highly associated with PROS1, including CD22 mediated by regulation,immunoregulatory-Internation, chemokine receptors bind chemokines, disease of the immune system, chemokine signaling pathway, IL-18 signaling pathway in glioma (**Figures 3E–L**). All In all, it is suggested from the above results that that PROS1 played an essential role in regulation of immune response in glioma.

### Correlations between PROS1 expression and tumor-infiltrating immune cells

**Figure 4:**
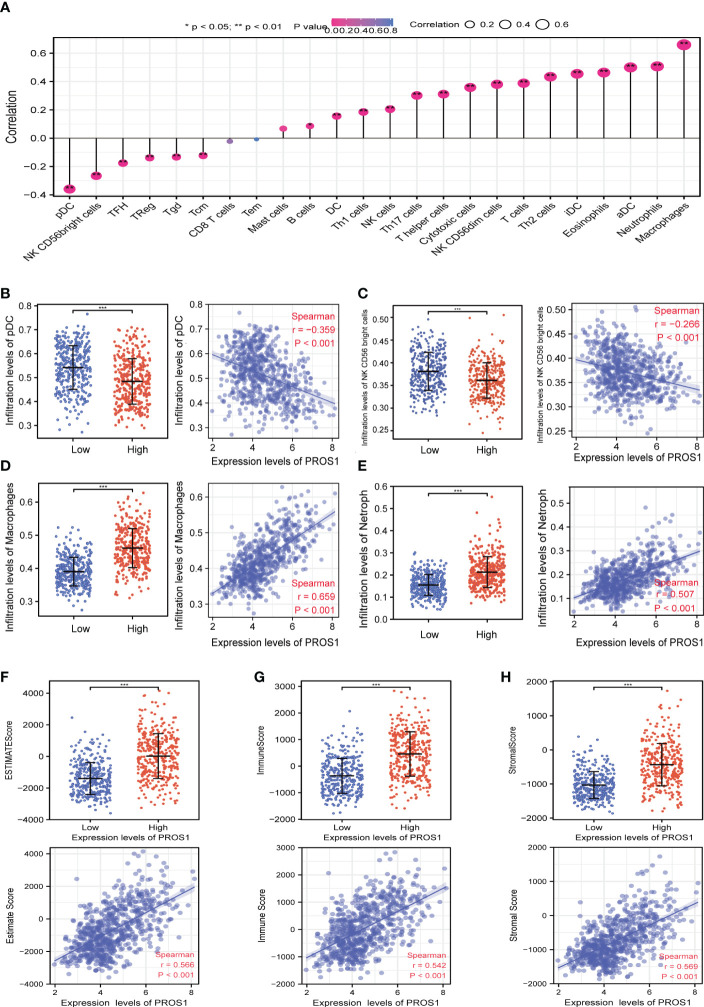
The relationship of immune cell infiltration and tumor immune microenvironment with PROS1 expression in GBM and LGG. **(A)** The relationship between PROS1 expression and the immune cell infiltration analyzed by ssGSEA in glioma. The PROS1 expression levels had a significant correlation with the infiltration of **(B)** pDCs, **(C)** NK CD56 Bright cells, **(D)** Macrophages, and **(E)** Neutrophils in glioma. The correlation of PROS1 expression level with **(F)** EstimateScore, **(G)** ImmuneScore, and **(H)** StromalScore in the high PROS1 and low PROS1 group in glioma. (*, *P* < 0.05; **, *P* < 0.01; ***, *P* < 0.001).

The lollipop chart showed the relationship between infiltration level of the 24 types of immune cells and expression level (TPM) of PROS1 (**Figure 4A**). It is found that levels of PROS1 expression were negatively correlated with the infiltration of pDCs, NK CD56 Bright cells (**Figures 4B, C**) and positively correlated with Macrophages and neutrophils in glioma (**Figures 4D, E**). As shown in **Figures 4F–H**, the high PROS1 group exhibited higher levels of the ImmuneScore, EstimateScore, and StromalScore than that of low PROS1 group in glioma. In conclusion, the above results showed that PROS1 is correlated with immune score and immune cell infiltration in glioma patients.

### PROS1 expression is associated with macrophages infiltration levels in LGG and GBM

**Figure 5:**
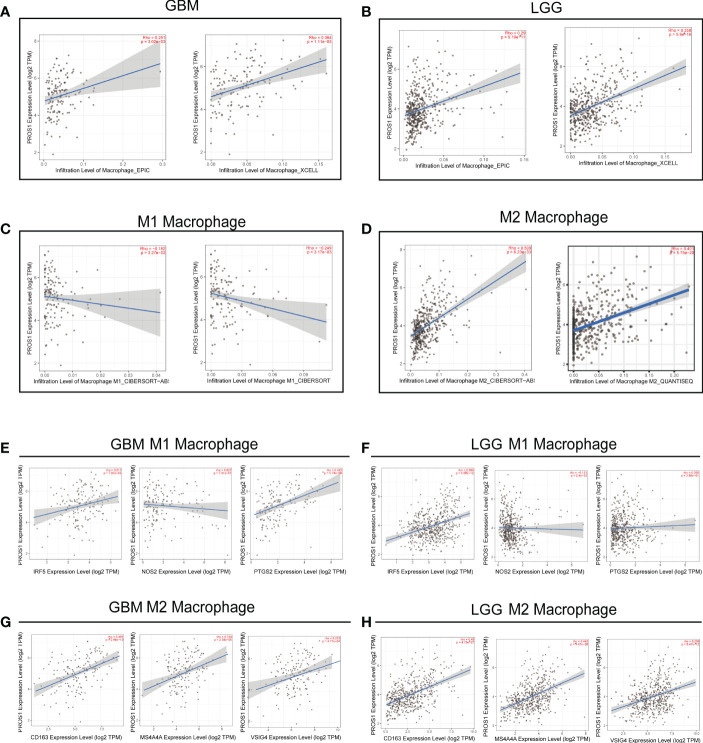
PROS1 expression correlates with marker sets of immune cells in GBM and LGG. **(A–D)** The correlation of PROS1 expression with Macrophages, M1 macrophages, and M2 macrophages infiltration level in glioma from the TIMER2.0 database. Scatter plots illustrate correlations between PROS1 expression and markers of M1 (IRF5, NOS2 and PTGS2) **(E, F)**, and M2 macrophages (CD163, MS4A4A, VSIG4) **(G, H)** in GBM and LGG.

Given that PROS1 expression has a remarkable correlation with the infiltration of macrophages, we further investigated the relationship of PROS1 expression with subtypes of macrophage (M1subtype and M2 subtype) infiltration by using the TIMER online tool. Our results indicated that expression of PROS1 was closely correlated with infiltration level of macrophage both in GBM and LGG **(** **Figures 5A, B**). Furthermore, we found that the expression of PROS1 was negatively correlated with infiltration of M1 macrophage while positively associated with infiltration of M2 macrophage in glioma **(** **Figures 5C, D**). Meanwhile, we analyzed the relationship between PROS1 and the marker sets of M1 phenotype and M2 phenotype in GBM and LGG. The results showed the expression of PROS1 has no significant relationship with IRF5, NOS2, and PTGS2 of M1 macrophages **(** **Figures 5E, F**). However, the scatter plots showed that the expression of PROS1 was positively associated with CD163, MS4A4A, and VSIG4 of M2 macrophages in GBM **(** **Figures 5G, H**). The above data indicated that increased PROS1 may play a vital role in decreasing the infiltration of M1 macrophages and inducing M2 macrophage infiltration and polarization in glioma.

### PROS1 expression is correlated with the immunosuppressive microenvironment

**Figure 6:**
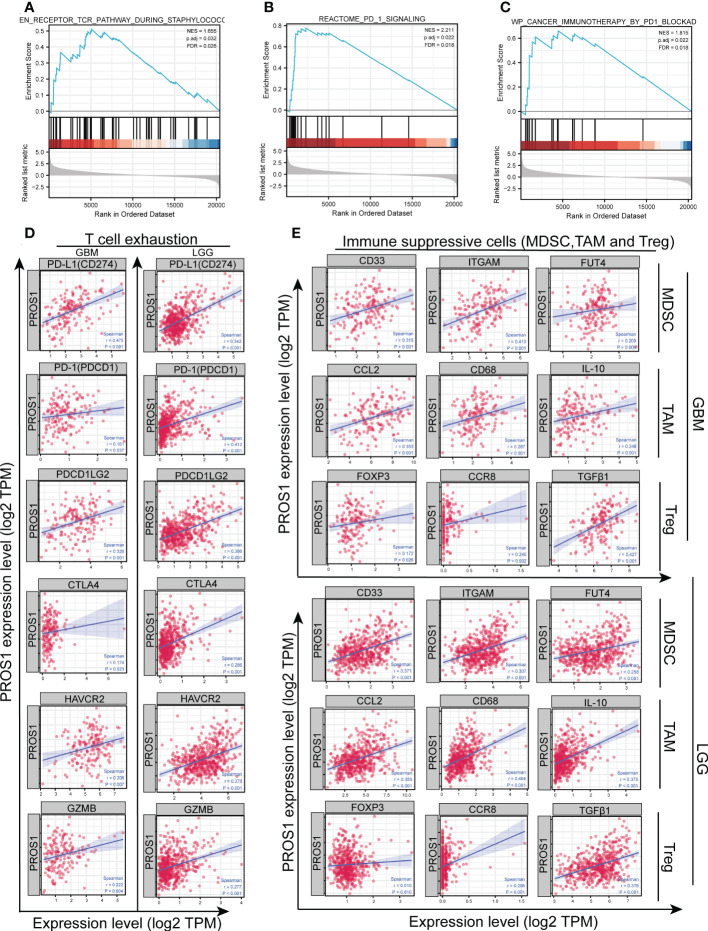
PROS1 expression is significantly correlated with the markers of immunosuppressive molecules in glioma. Enrichment plots from gene set enrichment analysis (GSEA) showed the high expression of PROS1 associated with **(A)** T cell antigen receptor pathway, **(B)** PD-1 signaling pathway, and **(C)** immunotherapy by PD1 blockade. **(D)** The Spearman correlation analysis revealed that the expression of PROS1 was correlated with the T cell exhaustion, including PD-L1, PD-1, PDCD1LG2, CTLA4, HAVCR2, and GZMB in glioma (all *P* < 0.05). **(E)** The Spearman correlation analysis results suggested the immune marker sets of MDSC (CD33, ITGAM, FUT4), TAM (CCL2, CD68, IL-10) and, Treg (FOXP3, CCR8, TGFβ1) were associated with the PROS1 expression in glioma.

In order to further investigate the possible mechanism of PROS1 on the impact of tumor immune micro-environment, GSEA analysis was conducted which revealed that high expression of PROS1 was correlated tothe T cell antigen receptor pathway **(** **Figure 6A**), the PD-1 signal pathway **(** **Figure 6B**) and the immunotherapy mediated by programmed cell death protein 1 (PD-1) blockade (**Figure 6C**). Then, we continued to explore the correlation between PROS1 expression and immunosuppressive molecules and immune checkpoints. It is found that the expression of PROS1 had positive correlations with PD-1, PD-L1, PDCD1LG2, CTLA4, HAVCR2, and GZMB (all *P <*0.05) in glioma **(** **Figure 6D**). In addition, the immunosuppressive cells play a vital role in the tumor microenvironment, including Treg, TAM, and MDSC. The Spearman correlation analysis results suggested that the immune marker sets of Treg (FOXP3, CCR8, TGFβ1), TAM (CCL2, CD68, IL-10), and MDSC (CD33, ITGAM, FUT4) were significantly associated with the PROS1 expression in glioma (**Figure 6E**). Therefore, the study showed that the increased PROS1 may promote tumor immune escape.

### Correlation of PROS1 expression with clinicopathological parameters in glioma patients

**Figure 7:**
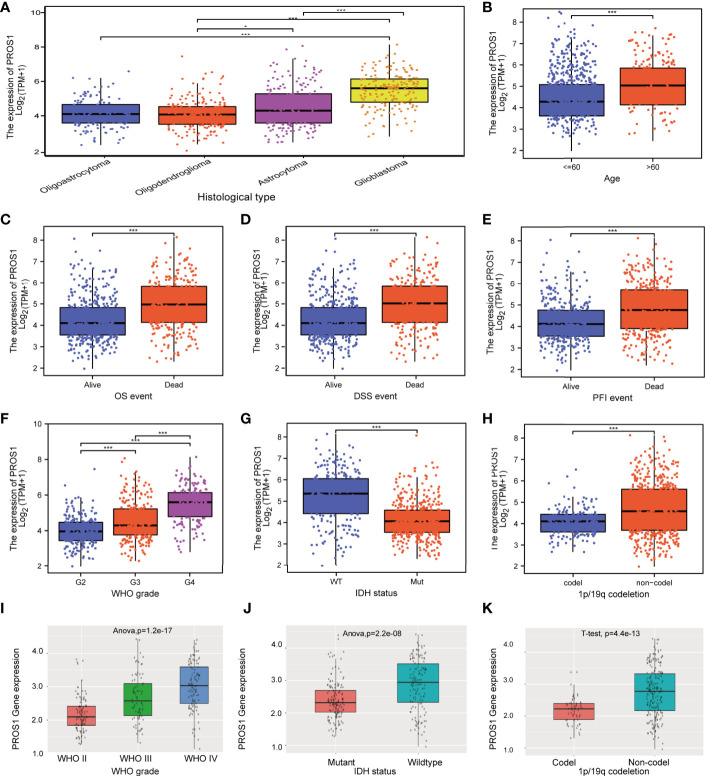
Relationship between PROS1 expression and clinical glioma parameters in the CGGA and TCGA cohorts. Association with PROS1 expression and clinicopathological characteristics, including **(A)** histological type, **(B)** age, primary therapy outcome **(C)** OS event, **(D)** DSS event, **(E)** PFI event, **(F)** WHO Grade, **(G)** IDH status, **(H)** 1p/19q codeletion, in TCGA. **(I–K)** The association between PROS1 expression and clinicopathological characteristics including WHO grade, IDH status, and 1p/19q codeletion in the CGGA Dataset (id mRNA_seq_325). *, *P* < 0.05; ***, *P* < 0.001.

In order to confirm the significance of PROS1 in glioma, we discussed the correlation between PROS1 expression and clinicopathological parameters in glioma patients. We analyzed 696 glioma patients with completed patient characteristics in TCGA. As shown in **Figures 7A–H** and **Table S1**, Increased PROS1 was closely associated with the histological type, age, outcome measures, WHO grade, IDH status, and 1p/19q codeletion(non-codel). In addition, PROS1 was found significantly increased in WHO IV, IDH-wildtype, and 1p/19q no-codeletion glioma patients in the CGGA cohort (id mRNA-seq_325) (**Figures 7I–K**). All the other clinical parameters evaluated were not significantly associated with PROS1 expression (**Table S1**). Also, the logistic regression analysis was applied to evaluate the correlation between the expression of PROS1 and clinical parameters (**Table S2**). All these findings proved that PROS1 expression could be associated with the malignant progression in glioma.

### PROS1 predicted worse survival in glioma

**Figure 8:**
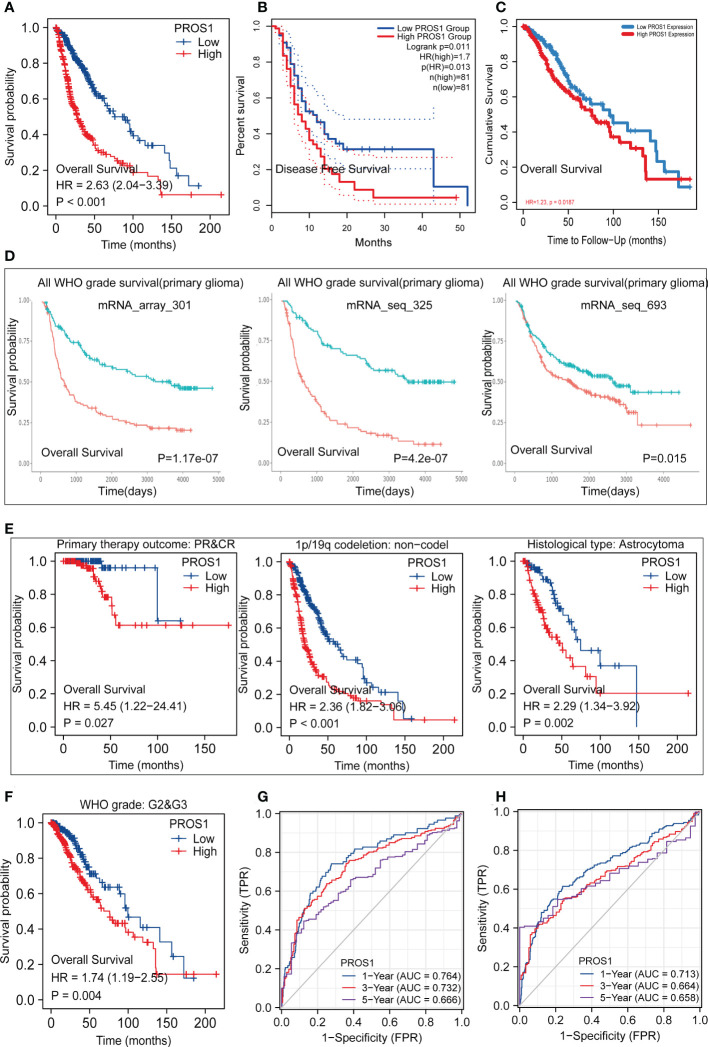
Kaplan-Meier survival and Receiver operating characteristic (ROC) curves. Kaplan-Meier survival analysis forglioma patients between PROS1-high and -low group in TCGA **(A)**, GEPIA2.0 **(B)**, TIMER2.0 **(C)**, CGGA **(D)**. **(E, F)** Subgroups Kaplan-Meier survival analysis of high and low PROS1 expression in PR&CR, 1p/19q codeletion, histological type, and G2&G3 glioma patients. **(G, H)** 1-, 3- and 5-year OS and PFI ROC curves based on risk score in TCGA.

Since the level of PROS1 was closely related to the progress of tumors in GBM and LGG, we subsequently discussed its prognosis significance. As shown in **Figure 8A**, Kaplan –Meier survival analysis using data collected from TCGA indicated that the high expression of PROS1 was closely correlated with poor OS in glioma. Similar results were acquired using data from the GEPIA, TIMER dataset, and CGGA datasets, increased PROS1 was correlated with poor DFS (**Figure 8B**) and OS (**Figures 8C, D**)in glioma. Subsequently, we displayed subgroup analyses of prognosis, which indicated that elevated PROS1 expression was correlated with poor overall survival in glioma patients with PR &CR,1p/19q non-codel, astrocytoma, and WHO G2&G3 grade (**Figures 8E, F**). Based on OS and PFI ROC curves of TCGA data, the relationship of PROS1 expression with 1-, 3-, and 5 years prognostic value were investigated. The results showed that PROS1 expression had a moderate prognostic value (**Figures 8G, H**). Thus, these part results showed that PROS1 could a possible prognostic marker for glioma patients.

### Prediction of the relationship between PROS1 expression and drug sensitivity

**Figure 9:**
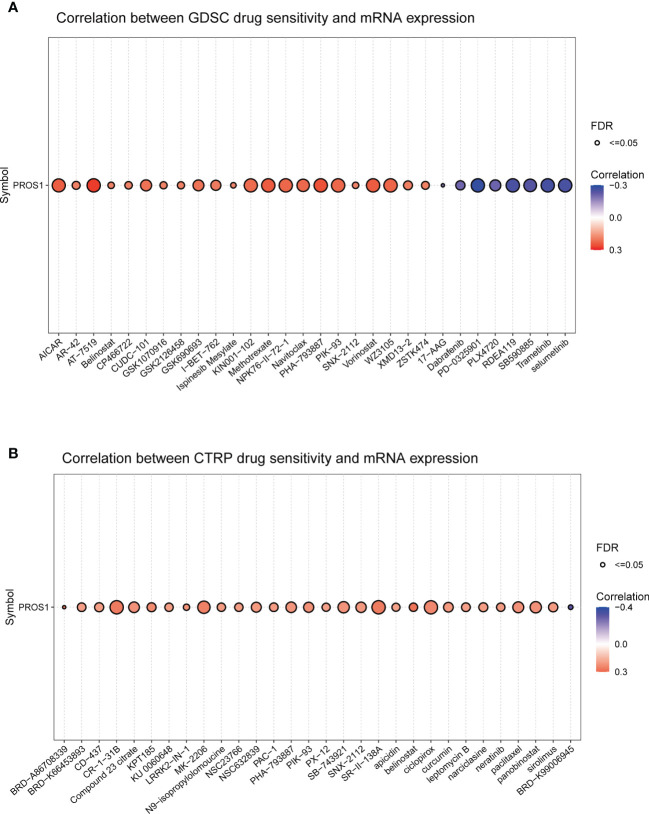
Correlation between PROS1 expression and the sensitivity of GDSC drugs and CTRP drugs (top 30) in pan-cancer **(A)** the correlation between PROS1 expression and the sensitivity of GDSC drugs (top 30) in pan-cancer **(B)** The association between PROS1 expression and the sensitivity of CTRP drugs (top 30) in pan-cancer.

The association between PROS1 expression and drug sensitivity was examined to identify its clinical significance using the Gene Set Cancer Analysis (GSCA) database. First, we explored the association between PROS1 expression and IC50 values of various molecules using the GDSC database. It is found that increased PROS1 was positively correlated with the IC50 values of small molecules, including AICAR, AT-7519, Methotrexate, Navitoclax, Vorinostat, and WZ3105, which suggested that increased PROS1 could lower the drug sensitivity for most small molecules (**Figure 9A**). Interestingly, high expression of PROS1 enhanced the sensitivity of eight drugs or small molecules, which included selumetinib, trametinib, SB590885, RDEA119, PLX4720, PD-0325901, Dabrafenib, 17-AAG (**Figure 9A**). Furthermore, we also used the CTRP database to explore the association between PROS1 and IC50 of various small molecules or drugs and found that increased PROS1 could lower the sensitivity of most small molecules, including CR-1-31B, MK-2206, ciclopirox and so on, except for BRD-K99006945 (**Figure 9B**). These results suggested that PROS1 was a potential cancer-resistant therapeutic target.

## Discussion

The present study comprehensively analyzed the expression of PROS1 in glioma, its prognostic value and its immune regulatory effect in TME, to provide potential strategies for glioma immunotherapy. In this study, we first explored the PROS1 gene expression by using multi-omics databases as well as *in vitro* experiments (qRT-PCR and Western Blotting). Moreover, we used single-cell analyses, GSEA analyses, GO,and KEGG pathway analyses to explore PROS1 functions in glioma. Subsequently, the relationship between PROS1 expression with immune infiltration and tumor immune microenvironment was analyzed in detail. As a result, increased PROS1 in glioma tissue could shape the micro-environment of immunosuppressive tumors to media its malignant progress.

The earlier study found that PROS1 expression was significantly higher in prostate cancer and which played a critical role in the progression of prostate cancer (18). Our multi-omics data results indicated that the expression of PROS1 in glioma had also increased significantly. Using quantitative PCR and western blot assay indicated that the protein and mRNA levels of PROS1 were significantly higher in glioma cells than in astroglia cells. The previous study also suggested that PROS1/TAM receptors were the effector of metastasis in the progress of papillary thyroid cancer progression (27). In addition, PROS1 was found to affect prostate cancer cell proliferation and resistance *via* regulating apoptosis (28). Consistent with the previous studies, through single -cell analysis and the results showed that PROS1 was closely correlated with invasion, metastasis, and proliferation of glioma cells. Furthermore, our wound healing and CCK8 assay results indicated that knockdown PROS1 expression could suppress U-87MG and LN-229 cell migration and proliferation *in vitro*. Taken together, PROS1 may promote the progression of glioma by affecting migration and proliferation. The other study reported that PROS1 could regulate immune response by activating the Tyro3 ligand (16) and increasing IFN-induced cytokine response (29). In our research, through the analysis of GSEA, KEGG, and GO pathways, the increased PROS1 was found to be associated with immune-related pathways, including immunoregulatory-internation, chemokine receptors bind chemokines, disease of the immune system, interactions between immune cells and chemokine signaling pathway. And PROS1 was identified to be involved in the regulation of the immune response in glioma. Nevertheless, there are few types of researches focusing on the immune regulation of PROS1 in glioma., and the relevant knowledge is relatively poor. Therefore, we furtherly comprehensive explored the relationship between PROS1 and immuno-modulatory in glioma.

More and more evidence shows that different proportions of immune infiltration cells are highly related to the state of immune response in antitumors (30, 31). At present, the molecules of targeted immune cell infiltration and checkpoint molecules can alter the effect of immunotherapy and affect the prognosis of cancer patients (30, 31). Using the TIMER online tool, increased PROS1 was observed to be significantly correlated with infiltrations of immune cells, including negatively associated with pDC, NK, and CD56 bright cells, and positively related with macrophages, and neutrophils in glioma. Penninger et al. explained that anticoagulant warfarin inhibited tumor metastasis *via* activating TAM receptors in NK cells (32). The decrease in NK cells may promote tumor metastasis and contribute to immunotherapy failure. In addition, Carrera Silva EA, et al. showed that PROS1could inhibit the activation of DC cells to restrain the immune response (33). Another study showed that tumor-associated macrophages constituted a significant proportion of the infiltrating immune cell and contributed to glioma progression in GBM (34, 35). Therefore, the significance of tumor-infiltrating macrophages in gliomas should be more thoroughly explored. Moreover, we found that PROS1 was strongly correlated with tumor-associated macrophages. Macrophages are mainly cataloged into M1 and M2 subtypes. In addition, M1 macrophages are anti-tumor surface, which plays a role in inhibiting tumor progress (36), while M2-activated macrophages promote tumor progress by secreting CD163 and other vascular generating factors (37).

M2-polarized macrophages lead to further suppression of the tumor immunosuppressive microenvironment (TIME) by secreting cytokines that inhibit T cells and other immune cell types (38). Christopher J. Kaler et al. suggested that membrane-bound PROS1 on tumor cells interacts with MERTK on nearby macrophages, leading to phosphorylation of MERTK and activation of downstream signaling that promotes M2 polarization in uveal melanoma (36). Similarly, it had been reported previously that M2 macrophage polarization following MerTK activation by its ligand PROS1 stimulation, which promotes cell migration and contributes to chemoresistance (39).Moreover, it is reported that the polarization from the M2 auxiliary type to the M1-yield can eliminate the restraint of immunosuppression, cause cytotoxic T cell immunity, and improve the efficacy of chemotherapy (40). Meanwhile, PROS1 was reported to be combined with Mer/Tyro3 receptor and effectively inhibit gene expression related to macrophage M1 (14, 41). In our research, we found PROS1 expression was significantly negatively correlated with the maker gene of M1 macrophage (NOS2), and positively related with maker genes of M2 macrophage (CD163, MS4A4A, VSIG4) both in GBM and LGG (**Figure 5**). Both of these results demonstrated that, in addition to increasing immune cell infiltration, high PROS1 expression could also induce macrophage M2 polarization that may induce tumor. Thus, the inhibition of PROS1 may represent a potential novel strategy to block macrophages to M2 phenotype polarization, and suppress glioma progression.

In addition, some studies revealed the effects of PROS1 expression on the tumor immunosuppressive microenvironment (41, 42). Also, it’s reported that targeted PROS1 could enhance the efficacy of anti-PD-1 therapy (43). Comparably, we observed that increased PROS1 had associations with the T cell antigen receptor pathway, PD-1 signaling pathway, and immunotherapy by PD1 blockade by GSEA analysis (**Figures 6A–C**). Glioma is called a “cold” tumor with poorly T cell-exhausted and less responsive to immune checkpoints (44). However, some studies have shown that targeted PROS1 might decrease immune checkpoint gene expression and increase the efficacy of immunotherapy (45). According to us, the expression of PROS1 is significantly related to marker genes of T cell exhaustion (PD-L1, HAVCR2, and CTLA4), and immunosuppressive immune cells (TAMs, MDSC, and Treg) (**Figures 6D, E**). Based on the above data, it could be considered that PROS1 induce glioma immunosuppression through T cell exhaustion, PD-L1 up-regulation, Treg and MDSC accumulation.

It is worth noting that high PD-L1 promoter methylation levels, and low tumor-infiltrating CD8 T lymphocyte cells may contribute to the failure of immunological treatment in IDH-mutant patients (46). Meanwhile, in a large randomized clinical trial (EORTC study 26053-22054), patients with co-deleted 1p/19q gliomas showed lower cancer-related mortality compared with those with non-co-deleted 1p/19q gliomas (47). In addition, increased PROS1 was associated with poor overall survival in glioma patients with IDH-Mut type, WHO G2&G3 grade, PR &CR,1p/19q non-codel, and astrocytoma. Finally, according to GDSC and CTRP databases, high PROS1 expression could reduce the drug sensitivity of small molecules, including AICAR, AT-7519, selumetinib, trametinib, SB590885, etc. Taken together, PROS1 is an independent risk factor for poor prognosis of glioma, which could shape the immune-suppressive tumor microenvironment to regulate the malignant progress of glioma, and may become a potential anti -cancer treatment target for patients with glioma.

This study has several limitations. Firstly, the bioinformatics research in our study focuses mostly on LGG and GBM, and more tumor types of glioma (such as astrocytoma, ependymomas, et al) need to be explored in the future. Secondly, further verification by a large number of clinical samples and clinical randomized controlled trials are still required in the future. Thirdly, *in vivo* experiments were not conducted because of the limitations of the laboratory conditions. The specific mechanism of PROS1 in promoting tumor progression and immune-suppressive should be further explored in future studies.

## Conclusion

In conclusion, this study clarifies high PROS1 expression with the malignant phenotype and poor prognosis of glioma. Moreover, our research also reveals the mechanism of PROS1 to promote the malignant progress by shaping the immune-suppressive tumor microenvironment, such as T cell failure, immunosuppressive cell infiltration, and M1 macrophages to the polarization of type M2 macrophages in glioma. Collectively, PROS1 can generally be used as promising biomarkers and potential immunotherapy targets.

## Data availability statement

The original contributions presented in the study are included in the article/**Supplementary Material**. Further inquiries can be directed to the corresponding authors.

## Ethics statement

TCGA and GEO belong to public databases. The patient participants involved in the database in accordance with the local legislation and institutional requirements. Users can download relevant data for free for research and publish relevant articles. Our study is based on open source data, so there are no ethical issues and other conflicts of interest.

## Author contributions

CY, LH designed and supervised the study. LH and WL designed the experimental scheme. JW, NW, XF, YL, and MH collected, analyzed, and visualized the data. JW and NW wrote the manuscript and performed cell experiments. LH, WL, and CY revised the manuscript. LH provided support for publishing funds. All authors contributed to the article and approved the submitted version.
