# Supplementary material for: PROS1 shapes the immune-suppressive tumor microenvironment and predicts poor prognosis in glioma
Source: Front Immunol. 2023 Jan 4;13:1052692. doi: 10.3389/fimmu.2022.1052692 (PMC9845921; doi:10.3389/fimmu.2022.1052692)
Supplement: Supplementary file 1 [file Table_1.docx]

Table S1. The correlation between clinicopathological variables and PROS1 expression

| Characteristic | Low expression of PROS1 | High expression of PROS1 | p |
| --- | --- | --- | --- |
| n | 348 | 348 |  |
| WHO grade, n (%) |  |  | < 0.001 |
| G2 | 154 (24.3%) | 70 (11%) |  |
| G3 | 131 (20.6%) | 112 (17.6%) |  |
| G4 | 24 (3.8%) | 144 (22.7%) |  |
| IDH status, n (%) |  |  | < 0.001 |
| WT | 59 (8.6%) | 187 (27.3%) |  |
| Mut | 287 (41.8%) | 153 (22.3%) |  |
| 1p/19q codeletion, n (%) |  |  | < 0.001 |
| codel | 118 (17.1%) | 53 (7.7%) |  |
| non-codel | 229 (33.2%) | 289 (41.9%) |  |
| Primary therapy outcome, n (%) |  |  | 0.067 |
| PD | 58 (12.6%) | 54 (11.7%) |  |
| SD | 89 (19.3%) | 58 (12.6%) |  |
| PR | 42 (9.1%) | 22 (4.8%) |  |
| CR | 94 (20.3%) | 45 (9.7%) |  |
| Gender, n (%) |  |  | 0.939 |
| Female | 150 (21.6%) | 148 (21.3%) |  |
| Male | 198 (28.4%) | 200 (28.7%) |  |
| Race, n (%) |  |  | 0.349 |
| Asian | 4 (0.6%) | 9 (1.3%) |  |
| Black or African American | 16 (2.3%) | 17 (2.5%) |  |
| White | 324 (47.4%) | 313 (45.8%) |  |
| Age, n (%) |  |  | < 0.001 |
| <=60 | 298 (42.8%) | 255 (36.6%) |  |
| >60 | 50 (7.2%) | 93 (13.4%) |  |
| Histological type, n (%) |  |  | < 0.001 |
| Astrocytoma | 103 (14.8%) | 92 (13.2%) |  |
| Glioblastoma | 24 (3.4%) | 144 (20.7%) |  |
| Oligoastrocytoma | 89 (12.8%) | 45 (6.5%) |  |
| Oligodendroglioma | 132 (19%) | 67 (9.6%) |  |
| OS event, n (%) |  |  | < 0.001 |
| Alive | 256 (36.8%) | 168 (24.1%) |  |
| Dead | 92 (13.2%) | 180 (25.9%) |  |
| DSS event, n (%) |  |  | < 0.001 |
| Alive | 261 (38.7%) | 170 (25.2%) |  |
| Dead | 80 (11.9%) | 164 (24.3%) |  |
| PFI event, n (%) |  |  | < 0.001 |
| Alive | 209 (30%) | 141 (20.3%) |  |
| Dead | 139 (20%) | 207 (29.7%) |  |
| Age, median (IQR) | 40 (32, 53) | 51 (38, 62) | < 0.001 |
