# Supplementary material for: PROS1 shapes the immune-suppressive tumor microenvironment and predicts poor prognosis in glioma
Source: Front Immunol. 2023 Jan 4;13:1052692. doi: 10.3389/fimmu.2022.1052692 (PMC9845921; doi:10.3389/fimmu.2022.1052692)
Supplement: Supplementary file 2 [file Table_2.docx]

**Table S2. PROS1 expression association with clinical-pathological characteristics (logistic regression)**

| Characteristics | Total(N) | Odds Ratio(OR) | P value |
| --- | --- | --- | --- |
| WHO grade (G3&G4 vs. G2) | 635 | 3.634 (2.580-5.157) | <0.001 |
| 1p/19q codeletion (non-codel vs. codel) | 689 | 2.810 (1.955-4.082) | <0.001 |
| IDH status (Mut vs. WT) | 686 | 0.168 (0.118-0.238) | <0.001 |
| Gender (Male vs. Female) | 696 | 1.024 (0.758-1.383) | 0.878 |
| Age (<=60 vs. >60) | 696 | 0.460 (0.312-0.671) | <0.001 |
| Histological type (Astrocytoma&Glioblastoma vs. Oligoastrocytoma&Oligodendroglioma) | 696 | 3.667 (2.687-5.030) | <0.001 |
| Race (White vs. Black or African American&Asian) | 683 | 0.743 (0.402-1.354) | 0.335 |
| Primary therapy outcome (PD&SD vs. PR&CR) | 462 | 1.547 (1.057-2.272) | 0.025 |
